# Association of perineural hydromorphone added to ropivacaine with early analgesic outcomes in proximal humerus fracture surgery: a retrospective cohort study

**DOI:** 10.3389/fmed.2026.1896305

**Published:** 2026-09-23

**Authors:** Peng Li, Qian Xu, Wei Liang, Kunhong Li, Changming Yang, Zhangqiang Chen

**Affiliations:** 1 Department of Anesthesiology, Wuhan Fourth Hospital, Wuhan, China; 2 Department of Anesthesiology, Jingmen Central Hospital, Jingmen City, China

**Keywords:** brachial plexus block, cortisol, hydromorphone hydrochloride, perioperative analgesia, peripheral nerve block adjuvant, proximal humerus fracture, retrospective cohort, ropivacaine

## Abstract

**Background:**

The analgesic benefit of adding perineural hydromorphone (HYD) to ropivacaine (ROP) for proximal humerus fracture surgery remains unclear.

**Objective:**

The aim of this study is to examine whether adding perineural hydromorphone to ropivacaine was associated with early analgesic outcomes and short-term adverse events.

**Methods:**

This single-center retrospective cohort included 130 consecutive eligible adults (ROP, *n* = 68; ROP + HYD, *n* = 62). The primary outcome was 12-h analgesia success, defined as resting visual analog scale (VAS) ≤3. We used multivariable logistic regression and propensity score analyses to account for measured treatment-preceding differences. Sensitivity analyses considered the combined block techniques, intraoperative medications, and postoperative analgesic co-interventions. A 1-point difference on the 0–10 VAS was used to interpret clinical importance.

**Results:**

Analgesia success at 12 h occurred in 39/62 patients (62.9%) in the ROP + HYD group and 26/68 (38.2%) in the ROP group [absolute difference 24.7 percentage points, 95% confidence interval (CI) 7.5–39.9; unadjusted odds ratio (OR) 2.74, 95% CI 1.35–5.57; number needed to treat 4.1]. The adjusted OR was 3.72 (95% CI 1.66–8.77), and propensity score analyses gave similar estimates. A sensitivity model that also accounted for superficial cervical plexus and T2 paravertebral field supplementation gave an OR of 3.83 (95% CI 1.67–9.31). Mean VAS differences favored ROP + HYD at 6 h (−0.33, 95% CI −0.65 to −0.02) and 12 h (−0.58, 95% CI −0.86 to −0.29), but both were smaller than 1 point; no difference was detected at 24 h. Median total 24-h rescue opioid consumption was 4.8 morphine milligram equivalents (MME) with ROP + HYD and 6.4 MME with ROP (*p* = 0.044). An unadjusted exploratory comparison found lower 6-h cortisol with ROP + HYD, but interpretation is limited by heterogeneous routine assay methods and multiple secondary comparisons. No between-group differences in the recorded short-term adverse events were detected.

**Conclusions:**

Adding perineural hydromorphone was associated with a higher proportion of patients achieving resting VAS ≤3 at 12 h. The average VAS differences were modest and no difference was detected at 24 h. Because treatment was not randomized, these findings should be confirmed in a prospective trial.

## 1 Introduction

Proximal humerus fractures account for approximately 5%–6% of all fractures and occur predominantly in older adults, in whom treatment is complicated by frailty, osteoporosis, and medical comorbidity (1, 2). Surgery can be followed by substantial early pain. Ultrasound-guided brachial plexus block, most often through an interscalene approach for shoulder and proximal humeral procedures, is therefore commonly incorporated into multimodal analgesia; however, pain may intensify when a single-injection block resolves (3, 4).

Ropivacaine (ROP) is a long-acting amide local anesthetic with a comparatively favorable cardiovascular and central nervous system toxicity profile, but the duration of a single injection may not cover the whole first postoperative day (5). Adjuvants have consequently been studied as a means of extending analgesia. The strongest evidence concerns dexamethasone and dexmedetomidine, both of which can prolong brachial plexus block, although effect estimates vary by drug, route, dose, and study quality (6, 7).

Evidence for opioid adjuvants is less settled. A broad review of peripheral nerve block adjuvants found marked heterogeneity across agents and procedures and did not support treating opioids as a single interchangeable class (8). Hydromorphone (HYD) is a potent *μ*-opioid receptor agonist, but direct evidence for its perineural use in brachial plexus block is sparse. A recent randomized trial of hydromorphone added to ropivacaine for serratus anterior plane block reported improved early postoperative analgesia, but findings from that interfascial-plane setting cannot be assumed to predict efficacy or safety in brachial plexus block (9).

Peripheral opioid receptors on primary afferent neurons provide a biologically plausible route for local analgesia, particularly in inflamed tissue, but systemic absorption may also contribute after perineural administration (10, 11). We therefore conducted a retrospective cohort study to examine whether adding hydromorphone hydrochloride 4 µg/kg to 0.375% ropivacaine was associated with analgesic outcomes and short-term adverse events in adults undergoing proximal humerus fracture surgery with ultrasound-guided brachial plexus block. The primary outcome was 12-h analgesia success, defined as resting visual analog scale (VAS) ≤3. This threshold is broadly compatible with published classifications of mild musculoskeletal pain, although it has not been specifically validated as a patient-acceptable threshold in this acute postoperative setting (12).

## 2 Materials and methods

### 2.1 Study design and setting

We conducted a single-center retrospective cohort study of consecutive adults who underwent proximal humerus fracture surgery with ultrasound-guided brachial plexus block between July 2024 and October 2025 at Wuhan Fourth Hospital. The exposure was the recorded block solution: 0.375% ropivacaine 20 mL alone or the same solution plus hydromorphone hydrochloride 4 µg/kg. Patients receiving both regimens were treated within this overall study interval, but ROP + HYD use was more concentrated in the later calendar period. Treatment choice reflected routine practice and attending anesthesiologist discretion; there was no random allocation or blinding. Temporal changes in clinical practice, operator experience, and perioperative care could therefore have influenced both treatment selection and outcomes. We interpret all treatment estimates as associations rather than causal effects.

### 2.2 Participants

Inclusion criteria: (i) age ≥18 years; (ii) unilateral proximal humerus fracture requiring open reduction and internal fixation (ORIF) or arthroplasty; (iii) ultrasound-guided brachial plexus block performed as the primary anesthetic; and (iv) availability of the data required to assess eligibility and the primary outcome.

Exclusion criteria: (i) bilateral/complex polytrauma; (ii) preexisting neurologic deficit of the operative limb; (iii) infection at the injection site, coagulopathy, or allergy to study drugs; (iv) conversion to general anesthesia before incision or failed block; (v) missing key outcomes; and (vi) other documented reasons for ineligibility.

Eligible patients were categorized by block solution into the following:

- ROP group: 0.375% ropivacaine 20 mL.
- ROP + HYD group: the same regimen plus perineural hydromorphone hydrochloride 4 µg/kg.

No *a priori* sample size calculation was performed because all patients meeting the analytic eligibility criteria during the study period were included. Based on the event rates observed in this cohort, a simple future two-group study would require approximately 64 evaluable patients per group to detect a difference of similar magnitude with 80% power at a two-sided *α* of 0.05. This planning estimate should be inflated for anticipated attrition and any additional design features; it does not replace a prospectively justified sample size calculation for a future trial.

### 2.3 Anesthesia protocol

Blocks were performed under real-time ultrasound guidance by attending anesthesiologists using 21- or 22-gauge echogenic needles. The interscalene approach was used in 116/130 patients (89.2%), with periplexus spread around the C5–C6/ upper trunk in the interscalene groove; 14/130 (10.8%) received a supraclavicular approach with spread around the trunks/divisions lateral to the subclavian artery. A superficial cervical plexus block was combined in 104 patients (80.0%), and 10 patients (7.7%) received documented T2 paravertebral field supplementation. The solution was injected incrementally with repeated negative aspiration.

Block adequacy was assessed 30 min after injection. Sensory block was tested by cold and pinprick over C5–C6 and the surgical field (0 = no loss, 1 = reduced sensation, 2 = absent sensation). Motor block was assessed by shoulder abduction and elbow flexion (0 = no block, 1 = mild weakness, 2 = marked weakness, 3 = complete block). Initial completion required adequate sensory and motor blockade at 30 min. Failure was defined as absence of adequate surgical field sensory loss by 30 min together with need for general anesthesia despite rescue. Delayed completion, supplemental targeted block, intravenous rescue for incision, and conversion to general anesthesia were recorded separately.

Procedural sedation was titrated clinically using midazolam, propofol, dexmedetomidine, and/or fentanyl; total doses were extracted and compared. Postoperative care followed defined institutional options rather than one uniform regimen. When intravenous patient-controlled analgesia (PCA) was used, the recorded sufentanil protocol was 1 µg/mL, 2 µg loading dose, 1.5 µg/h background infusion, 2 µg bolus, 15 min lockout, and 9.5 µg hourly maximum. Non-opioid analgesia included acetaminophen, flurbiprofen axetil, and celecoxib according to contraindications and clinician assessment. Rescue analgesia was administered for resting VAS >3 or patient request after bedside reassessment. All opioids were converted to morphine milligram equivalents (MME) and summarized for 0–6, 6–12, and 12–24 h.

### 2.4 Outcomes

#### 2.4.1 Primary outcome

The primary outcome was 12-h analgesia success, defined as resting VAS ≤3 on the 0–10 scale. The 12-h assessment was selected to represent the early postoperative period in which the effect of a single-injection block adjuvant would be expected to remain clinically relevant while breakthrough pain may begin to emerge. The VAS ≤3 threshold was selected as a patient-level responder definition because it is broadly compatible with published classifications of mild pain and aligned with the institutional rescue analgesia threshold of resting VAS >3 or patient request. It has not, however, been specifically validated as a patient-acceptable symptom-state threshold after proximal humerus fracture surgery; we therefore interpreted the responder analysis together with continuous VAS differences and a one-point threshold for clinical importance (12, 15).

#### 2.4.2 Secondary outcomes

(1) Resting VAS at 6, 12, and 24 h.
(2) Intraoperative hemodynamics: minimum mean arterial pressure (MAP); cumulative minutes with MAP <65 mmHg; number of hypotension episodes; minimum heart rate (HR); and vasopressor use (yes/no) and total dose if applicable.
(3) Serum biomarkers obtained during routine care: cortisol measured before the block and approximately 6 h afterward, and postoperative catecholamines (epinephrine, norepinephrine, and dopamine). The recorded laboratory assay method was chemiluminescent immunoassay (CLIA) or enzyme-linked immunosorbent assay (ELISA).
(4) Adverse events within 24 h: postoperative nausea and vomiting (PONV), pruritus, oversedation, hypoxia, urinary retention, and short-term neurologic symptoms.

Because this was a retrospective study without a prospectively registered statistical analysis plan, the outcome hierarchy should be interpreted as a retrospective analytic classification rather than as prospective prespecification. The 12-h responder endpoint was designated as the primary outcome for the comparative analysis; the remaining pain, hemodynamic, biomarker, and adverse-event measures were classified as secondary outcomes. Propensity score, combined block, and co-intervention models were used as sensitivity analyses.

### 2.5 Data collection and definitions

Data were extracted independently from electronic medical records, regional anesthesia notes, the anesthesia information system, medication administration records, PCA pump records, and laboratory records by two investigators using a standardized form. Treatment-preceding variables included demographics, American society of anesthesiologists (ASA) class, preoperative pain and opioid use, calendar period, surgery type, anesthesiologist, block approach, and operative variables. Intraoperative variables including midazolam, propofol, dexmedetomidine, and fentanyl doses were recorded. Postoperative variables included PCA parameters and delivered dose, acetaminophen, flurbiprofen axetil and celecoxib use, rescue analgesia timing, number of rescue doses, and MME by interval.

### 2.6 Statistical analysis

All analyses were performed in R with two-sided *α* = 0.05% and 95% confidence intervals (CIs). Continuous variables were summarized as mean (SD) when approximately symmetric and median interquartile range [IQR] when skewed; categorical variables were summarized as *n* (%). Between-group comparisons used independent-samples *t*-tests, Mann–Whitney *U*-tests, Pearson *χ*^2^ tests, or Fisher’s exact tests as appropriate. The primary effect was reported as an absolute risk difference, risk ratio, odds ratio, and number needed to treat (NNT), each with 95% CI where defined. The primary analysis focused on the 12-h responder outcome. No multiplicity adjustment was applied to secondary outcomes; their *p*-values and confidence intervals should therefore be interpreted as exploratory.

The multivariable logistic model included age, preoperative resting VAS, calendar period, preoperative opioid use within 30 days, and block approach. These variables were selected on clinical grounds and were measured before treatment. Propensity scores were estimated using age, sex, body mass index (BMI), ASA class, surgical side, smoking, diabetes, preoperative VAS, calendar period, surgery type, anesthesiologist, recent and chronic opioid use, and block approach. One-to-one nearest-neighbor matching used a caliper of 0.2 SD, and balance was assessed using absolute standardized mean differences (SMD), with values <0.10 indicating acceptable balance (13, 14). Overlap weighting was also applied to the full cohort. A secondary model examined the broader covariate set used in the original analysis. Because T2 paravertebral field supplementation differed between groups, a separate technique sensitivity model added superficial cervical plexus block and T2 supplementation to the parsimonious adjustment set.

VAS scores at 6, 12, and 24 h were examined using time-specific mean differences and a linear mixed model with a treatment-by-time interaction. A 1-point difference on the 0–10 VAS was used as the threshold for clinical interpretation (15). Intraoperative medication doses and postoperative analgesic co-interventions were compared between groups. Rescue analgesia and PCA use occurred after the block and could reflect both pain severity and subsequent treatment; they were therefore excluded from the baseline propensity score model. An additional sensitivity model included intraoperative medication doses, PCA use, and rescue MME through 12 h. Scheduled VAS values were abstracted irrespective of whether rescue analgesia had already been given; these outcomes therefore describe pain under routine postoperative care, including rescue treatment. First-rescue time was defined for the 99 patients who received rescue analgesia and was treated as undefined, rather than as an ordinary missing value, for the 31 patients who did not require rescue within 24 h. The other analyzed variables were complete.

### 2.7 Ethics

The study protocol was reviewed and approved by the institutional ethics committee (KY2024-144-01), which granted a waiver of informed consent owing to the retrospective design, de-identified data extraction, and minimal risk. Reporting follows STROBE recommendations for observational studies (16).

Trial registration: Not applicable. This was a retrospective observational study of existing records and was not prospectively registered as a clinical trial.

## 3 Results

### 3.1 Baseline characteristics

Among 146 records assessed for eligibility, 16 were excluded: bilateral fracture or complex polytrauma (*n* = 3); preexisting neurologic deficit of the operative limb (*n* = 3); injection site infection, coagulopathy, allergy, or another contraindication (*n* = 2); failed block or conversion to general anesthesia before incision (*n* = 3); missing key outcomes (*n* = 4); and other reasons (*n* = 1). The final analytic cohort comprised 130 patients: 68 in the ROP group and 62 in the ROP + HYD group (Figure 1).

**Figure 1:**
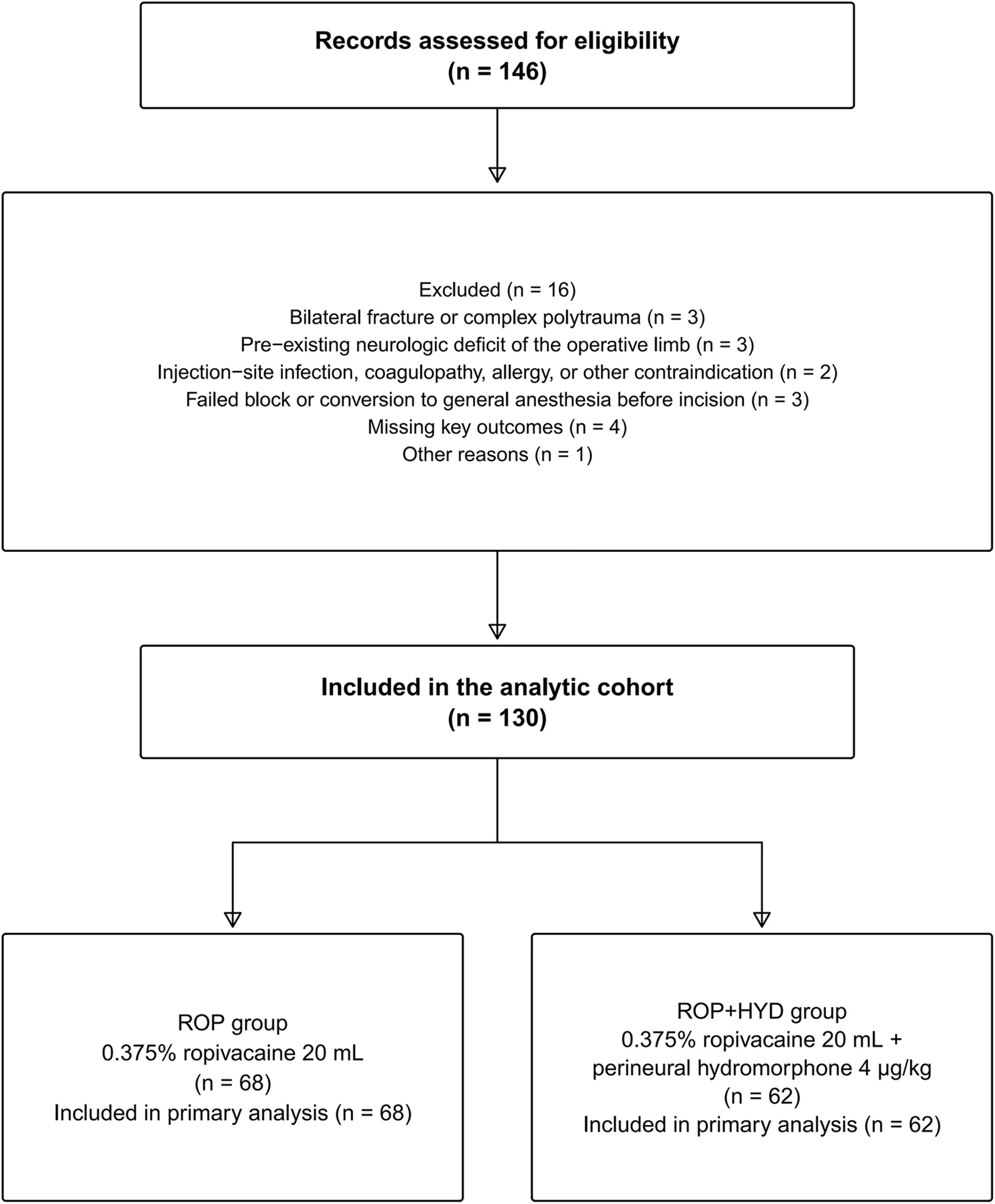
STROBE-style patient flow diagram. Of 146 records assessed for eligibility, 16 were excluded, leaving 130 patients in the analytic cohort: 68 in the ROP group and 62 in the ROP + HYD group. All 130 included patients were analyzed for the primary outcome. HYD, hydromorphone; ROP, ropivacaine.

Most baseline clinical characteristics were similar, but calendar period differed between groups (late period: 62.9% ROP + HYD vs. 32.4% ROP; *p* < 0.001). T2 paravertebral field supplementation was also more frequent with ROP + HYD [8/62 (12.9%) vs. 2/68 (2.9%); *p* = 0.047]. The main brachial plexus approach, superficial cervical plexus block, onset time, 30 min sensory and motor scores, initial block completion, supplemental block, incision rescue, and completion pathway did not differ detectably. Among the included patients, all 130 completed surgery without conversion to general anesthesia (Tables 1, 2).

**Table 1:** Baseline and treatment-preceding characteristics.

Variable	Level	Overall (*N* = 130)	ROP (*n* = 68)	ROP + HYD (*n* = 62)	*p*-value
Age, years		64.2 (12.5)	65.5 (12.1)	62.9 (12.9)	0.239
Sex	F	83 (63.8%)	40 (58.8%)	43 (69.4%)	0.212
	M	47 (36.2%)	28 (41.2%)	19 (30.6%)	
BMI, kg/m^2^		25.8 (3.3)	25.8 (3.1)	25.9 (3.6)	0.911
ASA physical status	1	6 (4.6%)	3 (4.4%)	3 (4.8%)	0.973
	2	63 (48.5%)	34 (50.0%)	29 (46.8%)	
	3	54 (41.5%)	27 (39.7%)	27 (43.5%)	
	4	7 (5.4%)	4 (5.9%)	3 (4.8%)	
Surgical side	Left	68 (52.3%)	33 (48.5%)	35 (56.5%)	0.366
	Right	62 (47.7%)	35 (51.5%)	27 (43.5%)	
Current smoker	0	92 (70.8%)	52 (76.5%)	40 (64.5%)	0.134
	1	38 (29.2%)	16 (23.5%)	22 (35.5%)	
Diabetes	0	105 (80.8%)	56 (82.4%)	49 (79.0%)	0.631
	1	25 (19.2%)	12 (17.6%)	13 (21.0%)	
Preoperative resting VAS		3.4 (1.4)	3.2 (1.3)	3.5 (1.4)	0.271
Calendar period	Early	69 (53.1%)	46 (67.6%)	23 (37.1%)	<0.001
	Late	61 (46.9%)	22 (32.4%)	39 (62.9%)	
Surgery type	Hemiarthroplasty	3 (2.3%)	1 (1.5%)	2 (3.2%)	0.705
	ORIF	118 (90.8%)	63 (92.6%)	55 (88.7%)	
	Reverse shoulder arthroplasty	9 (6.9%)	4 (5.9%)	5 (8.1%)	
Preoperative opioid use within 30 days	0	105 (80.8%)	57 (83.8%)	48 (77.4%)	0.355
	1	25 (19.2%)	11 (16.2%)	14 (22.6%)	
Chronic opioid use	0	127 (97.7%)	67 (98.5%)	60 (96.8%)	0.605
	1	3 (2.3%)	1 (1.5%)	2 (3.2%)	
Surgery duration, min		95.6 (24.5)	96.9 (24.9)	94.2 (24.2)	0.529
Estimated blood loss, mL		168 [105–232]	168 [101–222]	166 [128–241]	0.502
Pre-block MAP, mmHg		85.3 (10.2)	84.6 (10.0)	86.1 (10.5)	0.404
Pre-block HR, bpm		76.0 (13.0)	75.9 (13.8)	76.0 (12.2)	0.953

Values are mean (SD), median [IQR], or *n* (%). Calendar period was included in confounding-control analyses because it differed between groups.

**Table 2:** Block technique, objective assessment, and rescue pathway.

Variable	Level	Overall (*N* = 130)	ROP (*n* = 68)	ROP + HYD (*n* = 62)	*p*-value
Brachial plexus block approach	Interscalene	116 (89.2%)	63 (92.6%)	53 (85.5%)	0.188
	Supraclavicular	14 (10.8%)	5 (7.4%)	9 (14.5%)	
Combined superficial cervical plexus block	0	26 (20.0%)	17 (25.0%)	9 (14.5%)	0.136
	1	104 (80.0%)	51 (75.0%)	53 (85.5%)	
Other combined block	None	120 (92.3%)	66 (97.1%)	54 (87.1%)	0.047
	T2 paravertebral field supplementation	10 (7.7%)	2 (2.9%)	8 (12.9%)	
Needle type	21G × 50 mm echogenic	17 (13.1%)	9 (13.2%)	8 (12.9%)	0.346
	21G × 80 mm echogenic	3 (2.3%)	0 (0.0%)	3 (4.8%)	
	22G × 50 mm echogenic	99 (76.2%)	54 (79.4%)	45 (72.6%)	
	22G × 80 mm echogenic	11 (8.5%)	5 (7.4%)	6 (9.7%)	
Block onset time, min		14.4 (4.1)	14.3 (3.9)	14.5 (4.2)	0.771
Sensory score at 30 min	1	14 (10.8%)	8 (11.8%)	6 (9.7%)	0.701
	2	116 (89.2%)	60 (88.2%)	56 (90.3%)	
Motor score at 30 min	1	9 (6.9%)	6 (8.8%)	3 (4.8%)	0.627
	2	64 (49.2%)	34 (50.0%)	30 (48.4%)	
	3	57 (43.8%)	28 (41.2%)	29 (46.8%)	
Initial complete block at 30 min	0	14 (10.8%)	8 (11.8%)	6 (9.7%)	0.701
	1	116 (89.2%)	60 (88.2%)	56 (90.3%)	
Delayed completion before incision	0	124 (95.4%)	64 (94.1%)	60 (96.8%)	0.682
	1	6 (4.6%)	4 (5.9%)	2 (3.2%)	
Supplemental block	0	120 (92.3%)	62 (91.2%)	58 (93.5%)	0.747
	1	10 (7.7%)	6 (8.8%)	4 (6.5%)	
IV rescue analgesia for incision	0	126 (96.9%)	65 (95.6%)	61 (98.4%)	0.621
	1	4 (3.1%)	3 (4.4%)	1 (1.6%)	
Completion pathway	Delayed complete block before incision	6 (4.6%)	4 (5.9%)	2 (3.2%)	0.949
	Initial complete block	116 (89.2%)	60 (88.2%)	56 (90.3%)	
	IV rescue then regional anesthesia completion	2 (1.5%)	1 (1.5%)	1 (1.6%)	
	Supplemental block then success	6 (4.6%)	3 (4.4%)	3 (4.8%)	
Surgical anesthesia success	1	130 (100.0%)	68 (100.0%)	62 (100.0%)	NA
Conversion to general anesthesia	0	130 (100.0%)	68 (100.0%)	62 (100.0%)	NA

Sensory score: 0 = no loss, 1 = reduced, 2 = absent cold/pinprick. Motor score: 0 = no block, 1 = mild, 2 = marked, 3 = complete.

### 3.2 12 h analgesia success (resting VAS ≤3)

The ROP + HYD group achieved a higher 12-h analgesia success rate than ROP [39/62 (62.9%) vs. 26/68 (38.2%)]. The absolute difference was 24.7 percentage points (95% CI 7.5–39.9), corresponding to a risk ratio of 1.65 (95% CI 1.15–2.35), unadjusted odds ratio (OR) of 2.74 (95% CI 1.35–5.57), and NNT of 4.1 (95% CI 2.5–13.4; *p* = 0.005) (Table 3).

**Table 3:** Primary 12-h analgesia success effect measures.

Measure	Estimate	95% CI lower	95% CI upper	*p-*value
Control event rate	0.38	0.28	0.50	NA
Hydromorphone event rate	0.63	0.50	0.74	NA
Absolute risk difference	0.25	0.07	0.40	0.005
Risk ratio	1.65	1.15	2.35	0.005
Odds ratio	2.74	1.35	5.57	0.005
Number needed to treat	4.05	2.51	13.40	NA

Analgesia success was resting VAS ≤3. The number needed to treat was 4.1 (95% CI 2.5–13.4).

### 3.3 Adjusted and sensitivity analyses

The parsimonious multivariable model produced an adjusted OR of 3.72 (95% CI 1.66–8.77; *p* = 0.002). Propensity score matching retained 40 matched pairs and achieved absolute SMD <0.10 across modeled covariates; the matched OR was 3.86 (95% CI 1.47–10.10; *p* = 0.006). The overlap-weighted full-cohort estimate was 3.43 (95% CI 1.54–7.65; *p* = 0.003). T2 paravertebral field supplementation was more frequent in the ROP + HYD group, but adding this variable and superficial cervical plexus block to the parsimonious model produced a similar estimate (OR 3.83, 95% CI 1.67–9.31; *p* = 0.002). The broad covariate and co-intervention-adjusted models also yielded estimates in the same direction (Figure 2; Table 4).

**Figure 2:**
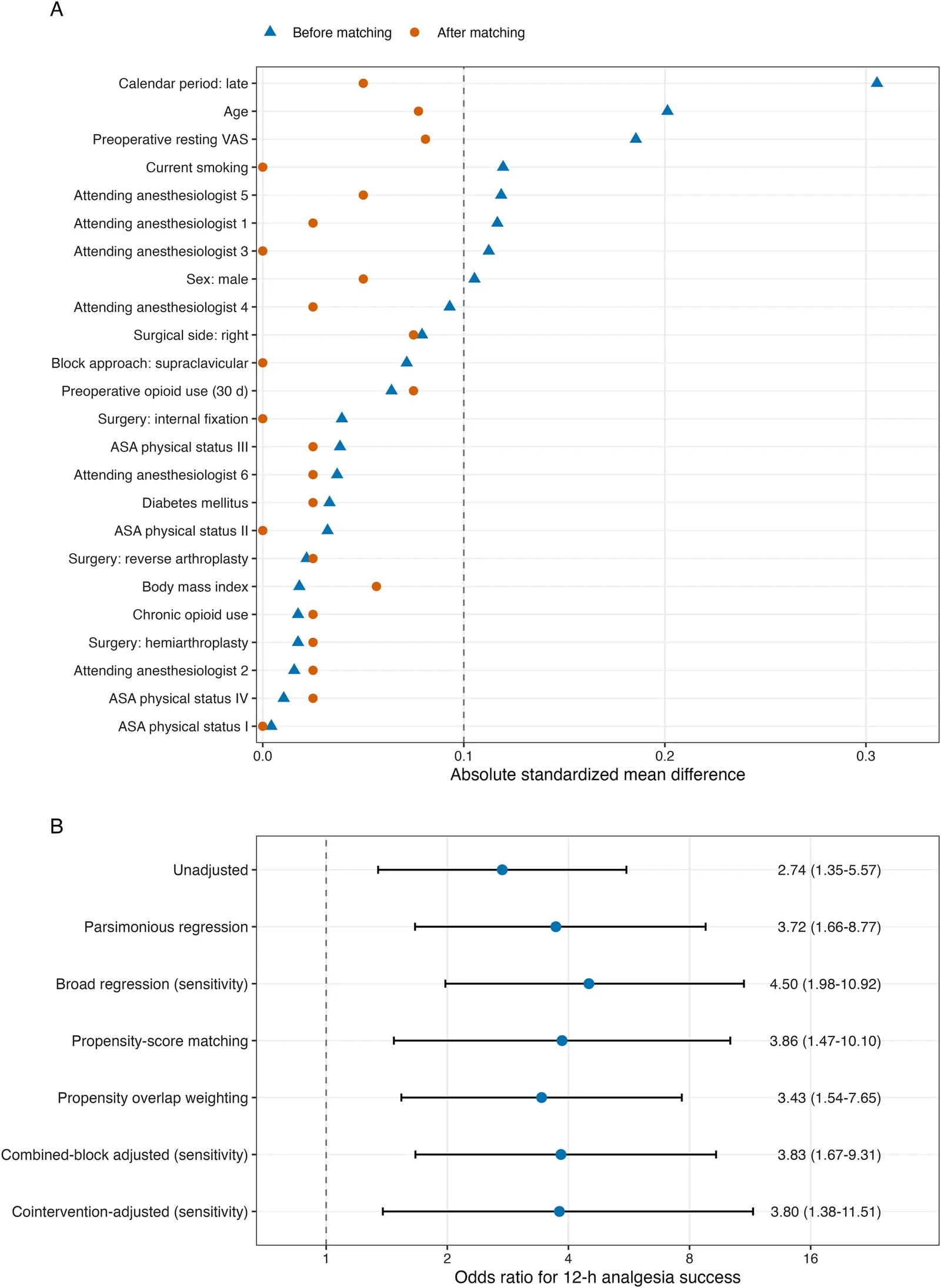
Confounding-control diagnostics and treatment effect sensitivity analyses for 12 h analgesia success. **(A)** Absolute standardized mean differences before and after 1:1 propensity score matching; the dashed line indicates 0.10. **(B)** Odds ratios and 95% confidence intervals across unadjusted, parsimonious outcome regression, broad adjustment, propensity-matched, overlap-weighted, combined-block-adjusted, and co-intervention-adjusted analyses.

**Table 4:** Treatment association across confounding-control analyses.

Analysis	Estimate	95% CI lower	95% CI upper	*p-*value
Unadjusted	2.74	1.35	5.57	0.005
Parsimonious covariate-adjusted	3.72	1.66	8.77	0.002
Broad covariate-adjusted sensitivity	4.50	1.98	10.92	<0.001
Propensity score matched	3.86	1.47	10.10	0.006
Propensity overlap-weighted	3.43	1.54	7.65	0.003
Combined-block-adjusted sensitivity	3.83	1.67	9.31	0.002
Co-intervention-adjusted sensitivity	3.80	1.38	11.51	0.013

Estimates are odds ratios with 95% CIs. The combined-block model adds superficial cervical plexus block and T2 paravertebral field supplementation to the parsimonious adjustment set. The co-intervention model is secondary because several included variables occur after treatment.

### 3.4 Resting VAS at 6, 12, and 24 h

Compared with ROP, the ROP + HYD group had lower mean resting VAS at 6 h (4.24 vs. 4.58; mean difference −0.33, 95% CI −0.65 to −0.02; *p* = 0.036) and 12 h (2.72 vs. 3.29; mean difference −0.58, 95% CI −0.86 to −0.29; *p* < 0.001), but neither difference reached the 1-point threshold used for clinical interpretation. At 24 h, the difference was −0.20 (95% CI −0.52 to 0.11; *p* = 0.209). Mixed-model contrasts were concordant (Table 5). At 12 h, VAS had been recorded after the first-rescue analgesic in 98 patients (75.4%), before first rescue in 1 (0.8%), and in 31 (23.8%) who did not require rescue within 24 h; this distribution was similar between groups (Table 6).

**Table 5:** Resting VAS effect sizes and the one-point threshold for clinical interpretation.

Time point	ROP mean	ROP SD	ROP + HYD mean	ROP + HYD SD	Mean difference	95% CI lower	95% CI upper	Standardized mean difference	*p*-value	Reaches 1-point threshold
6 h	4.58	0.96	4.24	0.83	−0.33	−0.65	−0.02	−0.37	0.036	No
12 h	3.29	0.78	2.72	0.85	−0.58	−0.86	−0.29	−0.71	<0.001	No
24 h	3.08	0.92	2.88	0.89	−0.20	−0.52	0.11	−0.22	0.209	No

Mean differences are ROP + HYD minus ROP. A 1-point difference on the 0–10 VAS was used as a threshold for clinical interpretation. This threshold and the outcome hierarchy were not prospectively registered.

**Table 6:** Intraoperative and postoperative analgesic co-interventions.

Variable	Level	Overall (*N* = 130)	ROP (*n* = 68)	ROP + HYD (*n* = 62)	*p-*value
Midazolam total dose, mg		1.3 [0.9–1.6]	1.2 [0.9–1.6]	1.3 [0.9–1.6]	0.952
Propofol total dose, mg		112.0 [95.5–132.0]	111.5 [94.0–136.0]	114.0 [97.2–130.0]	0.869
Dexmedetomidine total dose, µg		0.0 [0.0–26.0]	0.0 [0.0–28.5]	0.0 [0.0–20.8]	0.616
Fentanyl total dose, µg		60.0 [40.0–70.0]	60.0 [40.0–80.0]	60.0 [41.2–65.0]	0.182
Intravenous PCA used	0	41 (31.5%)	21 (30.9%)	20 (32.3%)	0.866
	1	89 (68.5%)	47 (69.1%)	42 (67.7%)	
PCA delivered during 24 h, µg		42.0 [0.0–49.8]	41.5 [0.0–48.2]	42.8 [0.0–49.9]	0.437
Acetaminophen used	0	36 (27.7%)	18 (26.5%)	18 (29.0%)	0.744
	1	94 (72.3%)	50 (73.5%)	44 (71.0%)	
Acetaminophen total 24 h, mg		2,000 [0–2,000]	2,000 [0–2,000]	2,000 [0–2,000]	0.510
Flurbiprofen axetil used	0	71 (54.6%)	36 (52.9%)	35 (56.5%)	0.688
	1	59 (45.4%)	32 (47.1%)	27 (43.5%)	
Flurbiprofen axetil total 24 h, mg		0 [0–100]	0 [0–100]	0 [0–100]	0.569
Celecoxib used	0	108 (83.1%)	57 (83.8%)	51 (82.3%)	0.812
	1	22 (16.9%)	11 (16.2%)	11 (17.7%)	
Celecoxib total 24 h, mg		0 [0–0]	0 [0–0]	0 [0–0]	0.816
Any rescue analgesia within 24 h	0	31 (23.8%)	16 (23.5%)	15 (24.2%)	0.929
	1	99 (76.2%)	52 (76.5%)	47 (75.8%)	
Number of rescue doses within 24 h among patients receiving rescue analgesia		1 [1–2]	1 [1–2]	1 [1–2]	0.212
Rescue opioid, 0–6 h, MME		3.2 [0.1–5.3]	3.8 [0.8–5.7]	2.4 [0.0–5.2]	0.199
Rescue opioid, 6–12 h, MME		0.5 [0.0–1.6]	0.8 [0.0–2.2]	0.3 [0.0–1.3]	0.076
Rescue opioid, 12–24 h, MME		0.2 [0.0–2.0]	0.2 [0.0–2.1]	0.0 [0.0–1.7]	0.210
Total rescue opioid, 0–24 h, MME		5.5 [1.1–7.8]	6.4 [1.8–8.7]	4.8 [0.5–6.8]	0.044
Time to first-rescue analgesia among patients receiving rescue analgesia, min		187.0 [143.5–254.0]	185.5 [124.5–258.2]	187.0 [144.5–249.5]	0.927
6-h VAS timing relative to first rescue	No rescue within 24 h	31 (23.8%)	16 (23.5%)	15 (24.2%)	0.341
	VAS measured after first rescue	93 (71.5%)	47 (69.1%)	46 (74.2%)	
	VAS measured before first rescue	6 (4.6%)	5 (7.4%)	1 (1.6%)	
12-h VAS timing relative to first rescue	No rescue within 24 h	31 (23.8%)	16 (23.5%)	15 (24.2%)	1.000
	VAS measured after first rescue	98 (75.4%)	51 (75.0%)	47 (75.8%)	
	VAS measured before first rescue	1 (0.8%)	1 (1.5%)	0 (0.0%)	
Major co-intervention	0	116 (89.2%)	59 (86.8%)	57 (91.9%)	0.342
	1	14 (10.8%)	9 (13.2%)	5 (8.1%)	

MME, morphine milligram equivalents; PCA, patient-controlled analgesia. Rescue variables were treated as post-exposure process/outcome variables and entered only in sensitivity analysis. Rescue count and first-rescue-time summaries are restricted to the 99 patients who received rescue analgesia; the 31 patients who required no rescue within 24 h are reported separately.

### 3.5 Perioperative co-interventions and intraoperative hemodynamics

Pre-block and intraoperative hemodynamic variables were similar between groups. Median total doses of midazolam (1.2 vs. 1.3 mg), propofol (111.5 vs. 114.0 mg), dexmedetomidine (0 vs. 0 µg), and fentanyl (60 vs. 60 µg) did not differ significantly (all *p* ≥ 0.18). PCA use and non-opioid analgesic use were also similar. Total 24-h rescue opioid consumption was lower in ROP + HYD (median 4.8 vs. 6.4 MME; *p* = 0.044), although rescue incidence and timing did not differ (Table 6).

Intraoperatively, the minimum MAP did not differ (72.1 ± 11.9 vs. 74.7 ± 12.0 mmHg, *p* = 0.215). Hypotension episodes (median [IQR]) were comparable (1.00 [0–3] vs. 2.00 [0–3], *p* = 0.266). Minimum HR was similar (67.8 ± 15.2 vs. 67.4 ± 14.0 bpm, *p* = 0.870), and the distribution of bradycardia episodes did not differ (0 episodes: 80.9% vs. 83.9%, *p* = 0.764). Vasopressor use was frequent and comparable (79.4% vs. 83.9%, *p* = 0.668) (Table 7).

**Table 7:** Clinical outcomes and short-term adverse events.

Variable	Level	Overall (*N* = 130)	ROP (*n* = 68)	ROP + HYD (*n* = 62)	*p*-value
Analgesia success at 12 h	0	65 (50.0%)	42 (61.8%)	23 (37.1%)	0.005
	1	65 (50.0%)	26 (38.2%)	39 (62.9%)	
Resting VAS at 6 h		4.42 (0.91)	4.58 (0.96)	4.24 (0.83)	0.036
Resting VAS at 12 h		3.02 (0.86)	3.29 (0.78)	2.72 (0.85)	<0.001
Resting VAS at 24 h		2.99 (0.91)	3.08 (0.92)	2.88 (0.89)	0.209
Minimum MAP, mmHg		73.4 (12.0)	72.1 (11.9)	74.7 (12.0)	0.215
Time with MAP <65 mmHg, min		7.0 [2.0–13.0]	6.0 [1.0–12.0]	8.0 [2.2–13.0]	0.273
Hypotension episodes		1.0 [0.0–3.0]	1.0 [0.0–3.0]	2.0 [0.0–3.0]	0.266
Minimum HR, bpm		67.6 (14.6)	67.8 (15.2)	67.4 (14.0)	0.870
Bradycardia episodes	0	107 (82.3%)	55 (80.9%)	52 (83.9%)	0.764
	1	16 (12.3%)	9 (13.2%)	7 (11.3%)	
	2	1 (0.8%)	0 (0.0%)	1 (1.6%)	
	3	6 (4.6%)	4 (5.9%)	2 (3.2%)	
Vasopressor used	1	106 (81.5%)	54 (79.4%)	52 (83.9%)	0.668
Pre-block cortisol, nmol/L		429 (113)	439 (123)	417 (102)	0.279
Cortisol at 6 h, nmol/L		495 (157)	523 (167)	465 (140)	0.034
Epinephrine, pg/mL		64.1 (23.9)	65.4 (24.6)	62.8 (23.3)	0.536
Norepinephrine, pg/mL		369.1 (109.4)	381.0 (101.7)	356.0 (116.6)	0.198
Dopamine, pg/mL		20.6 (8.6)	20.9 (9.2)	20.2 (8.0)	0.649
Biomarker assay method	CLIA	97 (74.6%)	56 (82.4%)	41 (66.1%)	0.044
	ELISA	33 (25.4%)	12 (17.6%)	21 (33.9%)	
PONV within 24 h	0	98 (75.4%)	52 (76.5%)	46 (74.2%)	0.763
	1	32 (24.6%)	16 (23.5%)	16 (25.8%)	
Pruritus within 24 h	0	118 (90.8%)	61 (89.7%)	57 (91.9%)	0.661
	1	12 (9.2%)	7 (10.3%)	5 (8.1%)	
Oversedation within 24 h	0	121 (93.1%)	62 (91.2%)	59 (95.2%)	0.497
	1	9 (6.9%)	6 (8.8%)	3 (4.8%)	
Hypoxia within 24 h	0	124 (95.4%)	64 (94.1%)	60 (96.8%)	0.682
	1	6 (4.6%)	4 (5.9%)	2 (3.2%)	
Urinary retention within 24 h	0	117 (90.0%)	64 (94.1%)	53 (85.5%)	0.101
	1	13 (10.0%)	4 (5.9%)	9 (14.5%)	
Transient neurologic symptoms	0	127 (97.7%)	67 (98.5%)	60 (96.8%)	0.605
	1	3 (2.3%)	1 (1.5%)	2 (3.2%)	

PONV, postoperative nausea and vomiting; VAS, visual analog scale; MAP, mean arterial pressure; HR, heart rate; CLIA, chemiluminescent immunoassay; ELISA, enzyme-linked immunosorbent assay. Biomarker and other secondary-outcome *p*-values are unadjusted for multiple comparisons and should be interpreted as exploratory. NA indicates that a between-group test was not applicable because the outcome was constant.

### 3.6 Serum cortisol and catecholamines

Baseline cortisol was comparable between ROP + HYD and ROP (417 ± 102 vs. 439 ± 123 nmol/L, *p* = 0.279). In an unadjusted exploratory comparison, 6-h cortisol was lower with ROP + HYD than ROP (465 ± 140 vs. 523 ± 167 nmol/L, *p* = 0.034). However, assay method distribution differed between groups: CLIA was used in 41/62 (66.1%) ROP + HYD patients and 56/68 (82.4%) ROP patients, whereas ELISA was used in 21/62 (33.9%) and 12/68 (17.6%), respectively (Fisher exact *p* = 0.044). Postoperative catecholamines were similar between groups: epinephrine 62.8 ± 23.3 vs. 65.4 ± 24.6 pg/mL (*p* = 0.536), norepinephrine 356 ± 117 vs. 381 ± 102 pg/mL (*p* = 0.198), and dopamine 20.2 ± 8.03 vs. 20.9 ± 9.15 pg/mL (*p* = 0.649). Because these were unadjusted secondary comparisons without multiplicity correction, they are exploratory (Table 7).

### 3.7 Short-term adverse events

No between-group differences were detected in PONV (25.8% ROP + HYD vs. 23.5% ROP; *p* = 0.763), pruritus (8.1% vs. 10.3%; *p* = 0.661), oversedation (4.8% vs. 8.8%; *p* = 0.497), hypoxia (3.2% vs. 5.9%; *p* = 0.682), urinary retention (14.5% vs. 5.9%; *p* = 0.101), or transient neurologic symptoms (3.2% vs. 1.5%; *p* = 0.605). Event counts were small (Table 7).

## 4 Discussion

In this retrospective cohort, 62.9% of patients given ROP + HYD achieved resting VAS ≤3 at 12 h, compared with 38.2% of those given ROP. The absolute difference was 24.7 percentage points and the corresponding NNT was approximately 4. Estimates from multivariable regression, propensity score matching, and overlap weighting pointed in the same direction. This consistency is reassuring with respect to the measured covariates, but it does not remove confounding from unmeasured clinical decisions or from the pronounced difference in calendar period distribution between groups. Although both regimens were used within the July 2024–October 2025 study interval, ROP + HYD use was more concentrated in the later period. Changes over time in operator experience, block practice, perioperative co-interventions, documentation, or other aspects of care may therefore have contributed to the observed association.

The findings should be viewed against a much larger evidence base for non-opioid adjuvants. Meta-analyses show that dexamethasone and dexmedetomidine can extend the duration of brachial plexus block, although the certainty and magnitude of benefit vary according to route and outcome (6, 7). By comparison, the evidence for perineural opioids is heterogeneous and agent specific (8). Direct clinical evidence for hydromorphone as a peripheral block adjuvant remains especially limited. The recent serratus anterior plane block trial reported less postoperative pain when hydromorphone was combined with ropivacaine (9), but differences in anatomy, local tissue uptake, surgery, and background analgesia prevent a direct comparison with our cohort. Our findings therefore provide a signal that warrants prospective evaluation in brachial plexus block rather than confirmation of an established treatment effect.

Two mechanisms could account for an early association. Opioid receptors expressed on peripheral sensory terminals can become more available and functionally active in inflamed tissue, and receptor activation may reduce nociceptor excitability and local inflammatory signaling (10, 11). This mechanism is plausible after fracture and surgery, but it was not measured here. Hydromorphone may also enter the systemic circulation from the injection site. Pharmacokinetic studies confirm that hydromorphone is systemically bioavailable and readily distributed after administration (17); without plasma concentrations or a systemic-hydromorphone comparison group, the present data cannot separate a local receptor-mediated effect from systemic analgesia.

The outcome pattern suggests that any association was concentrated in the early postoperative period. Mean resting VAS was lower with ROP + HYD at 6 and 12 h, and 24-h rescue opioid consumption was modestly lower, whereas no VAS difference was detected at 24 h. Rescue incidence and observed first-rescue timing did not differ between groups. Because block duration and time to sensory recovery were not recorded, the reason for the loss of between-group separation at 24 h remains uncertain. Future trials should standardize rescue treatment and scheduled pain assessment, distinguish measurements obtained before and after rescue, and prospectively measure time to first pain, sensory and motor block duration, rebound pain, cumulative opioid consumption, and recovery beyond 24 h.

The responder and continuous pain outcomes convey different aspects of benefit. The 24.7-percentage-point increase in patients reaching VAS ≤3 is easy to interpret clinically, but the between-group mean differences were only 0.33 points at 6 h and 0.58 points at 12 h. Both were below the one-point threshold used for clinical interpretation, consistent with published work on meaningful change in acute postoperative VAS scores (15). A threshold outcome can change appreciably when many observations lie close to the cut point, even when the average shift is small. Accordingly, the NNT should not be read as evidence of a large reduction in pain intensity for every patient.

The unadjusted 6-h cortisol comparison favored ROP + HYD, whereas baseline cortisol and postoperative catecholamines were similar. Surgery activates the hypothalamic–pituitary–adrenal and sympathoadrenal systems, and cortisol is influenced by the magnitude and timing of tissue injury, analgesia, anesthetic drugs, circadian rhythm, patient-level factors, and laboratory measurement method (18, 19). In this cohort, CLIA and ELISA were both used and their distribution differed between groups; moreover, multiple secondary outcomes were examined without multiplicity correction. The isolated cortisol result is therefore hypothesis-generating and is not specific evidence of an anti-inflammatory or stress-modulating action of perineural hydromorphone. Standardized assays, repeated biomarker measurements, and prespecified mediation analyses would be needed before drawing mechanistic conclusions.

Events such as PONV, pruritus, oversedation, hypoxia, urinary retention, transient neurologic symptoms, and hemodynamic outcomes did not differ detectably between groups. This is useful short-term descriptive information, but the absence of a statistically significant difference is not evidence of equivalence. Event counts were low, confidence intervals around uncommon harms would be wide, and follow-up ended at 24 h. The study cannot exclude rare respiratory events, delayed neurologic symptoms, neurotoxicity, or infection. Until adequately powered prospective safety data are available, hydromorphone should not be regarded as a routine perineural adjuvant on the basis of this cohort alone.

Several features strengthen the analysis: consecutive-case inclusion, a clearly defined primary outcome, detailed extraction of block technique and perioperative co-interventions, reporting of absolute and relative effects, and concordant estimates across several confounding-control approaches. However, important limitations remain. Treatment was selected by the attending anesthesiologist rather than randomized, and use of ROP + HYD increased in the later calendar period. T2 paravertebral field supplementation was also more common with ROP + HYD. Although a sensitivity model that included both combined block variables gave a similar estimate, only 10 patients received T2 supplementation, and residual confounding remains possible. Patients and clinicians were not blinded, so pain reporting and rescue treatment may have been influenced by knowledge of the block solution. Propensity methods balance measured variables only, and unrecorded changes in practice may remain (13, 14). Because no prospectively registered statistical analysis plan was available, the outcome hierarchy represents a retrospective analytic classification. Patients with failed blocks or conversion to general anesthesia before incision were excluded, so this cohort cannot compare overall block failure rates between solutions. The sample was small for safety assessment, one dose was studied at one center, block duration was not measured, and follow-up was limited to 24 h. Because most scheduled 12-h VAS assessments occurred after rescue analgesia, the primary outcome reflects pain under usual postoperative treatment rather than an untreated block effect; adjustment for rescue treatment is also post-exposure and cannot recover a causal direct effect. Biomarker analyses were further limited by heterogeneous assay methods and the absence of multiplicity correction. Because the cohort was restricted to adults undergoing proximal humerus fracture surgery with brachial plexus block at a single center, the findings should not be extrapolated directly to other surgical procedures, different peripheral or fascial-plane blocks, other hydromorphone doses, or patient populations with different clinical characteristics. The broad regression model also contained many parameters relative to the number of events and should be considered supportive rather than definitive.

These limitations define the next step. A prospective, randomized, double-blind, dose-ranging trial should compare ropivacaine alone and ropivacaine plus perineural hydromorphone, and, ideally, a systemic-hydromorphone control. It should prespecify both a patient-centered pain endpoint and an objective block duration endpoint, standardize multimodal analgesia, follow neurologic and respiratory safety beyond discharge, and report rebound pain and opioid-related adverse effects. Such a design would determine whether the early association observed here reflects a clinically worthwhile local adjuvant effect.

## 5 Conclusion

In this retrospective cohort, perineural hydromorphone added to ropivacaine was associated with a higher 12-h analgesia success rate. The mean VAS differences were smaller than 1 point, and no difference was detected at 24 h. Non-random treatment selection and residual confounding limit causal interpretation. A prospective randomized study is needed to determine efficacy, dose, and safety.

## Data Availability

The original contributions presented in the study are included in the article/Supplementary Material; further inquiries can be directed to the corresponding authors.
